# Supplementary figures and images for: Reverse vaccinology-based identification of a novel surface lipoprotein that is an effective vaccine antigen against bovine infections caused by Pasteurella multocida
Source: PLoS Pathog. 2023 Mar 24;19(3):e1011249. doi: 10.1371/journal.ppat.1011249 (PMC10075479; doi:10.1371/journal.ppat.1011249)

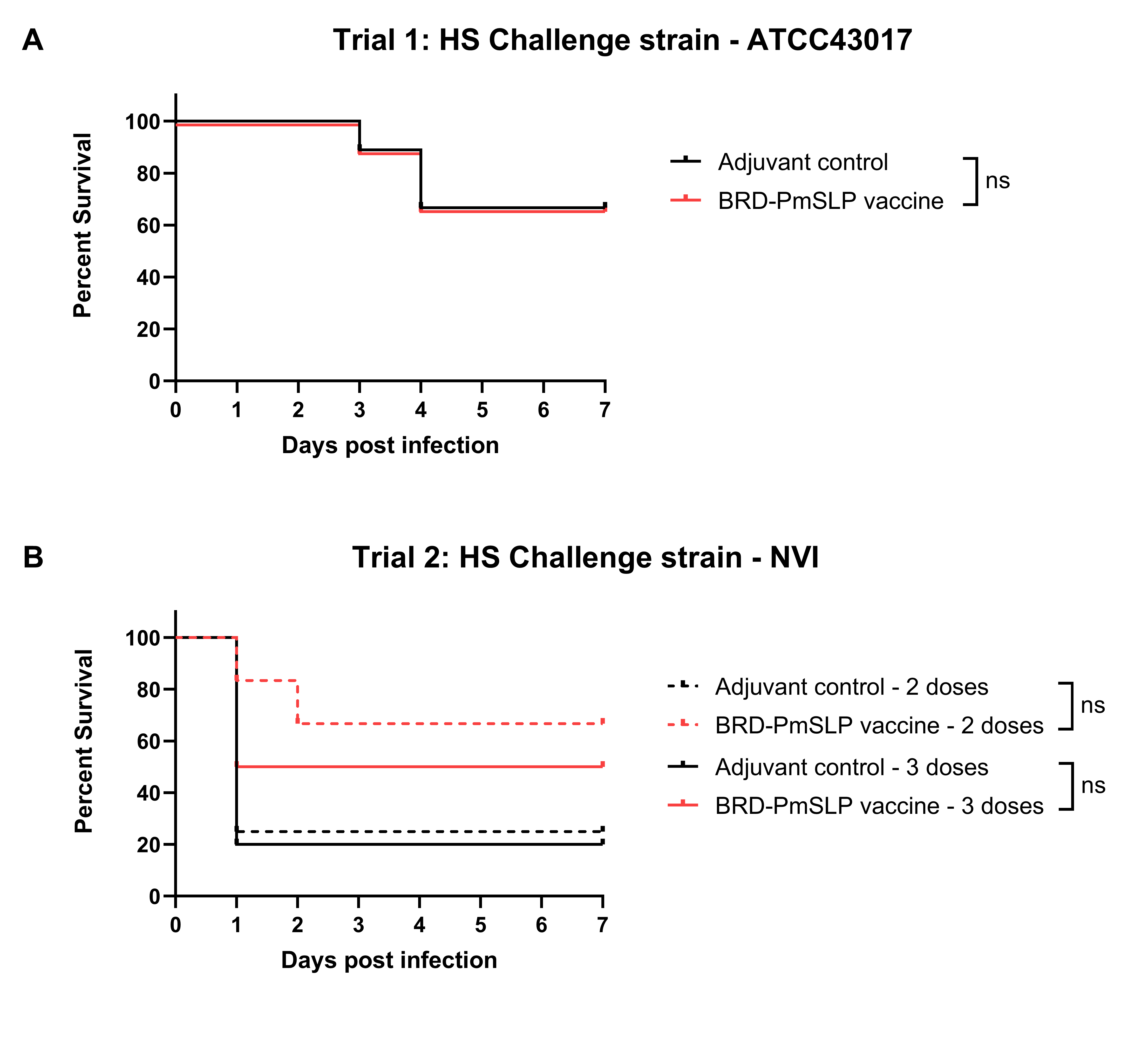

Supplement: S1 Fig — A. Survival of cattle immunized with three doses of BRD-PmSLP vaccine or adjuvant control following challenge with a South Asian Serogroup B HS isolate ATCC43017. N = 9 animals per group. B. Survival of cattle immunized with two or three doses of BRD-PmSLP vaccine or adjuvant control following challenge with an Ethiopian Serogroup B HS strain. N = 4–6 per group. Log-rank (Mantel Cox) tests performed for survival curve comparisons; ns, not significant. (TIF) [file ppat.1011249.s001.tif]
