# Supplementary material for: Reverse vaccinology-based identification of a novel surface lipoprotein that is an effective vaccine antigen against bovine infections caused by Pasteurella multocida
Source: PLoS Pathog. 2023 Mar 24;19(3):e1011249. doi: 10.1371/journal.ppat.1011249 (PMC10075479; doi:10.1371/journal.ppat.1011249)
Supplement: S2 Table — N denotes number of animals per group;–denotes not detected; + denotes detected; N/A denotes not applicable (no animals fell within this group); ND denotes no data. (PDF) [file ppat.1011249.s003.pdf]

Table S2: Bacterial recovery and detection of challenged animals. N denotes number of animals per group; – denotes not detected; + denotes detected; N/A denotes not applicable (no animals fell within this group); ND denotes no data.

**Formulation**	**HS-PmSLP +AlOH**		**HS-PmSLP +Gel02+Poly(I:C)**		**Bacterin +AlK(SO_4)$_2$**		**Adjuvant**	
**Outcome**	Alive	Dead	Alive	Dead	Alive	Dead	Alive	Dead
N	7	1	6	2	8	0	0	8
**Bacterial Recovery**								
Blood	-	+	-	+	-	N/A	N/A	+
**Molecular Detection**								
Blood	-	+	-	+	-	N/A	N/A	+
Lung	ND	+	ND	+	ND	N/A	N/A	+
Liver	ND	+	ND	+	ND	N/A	N/A	+
Kidney	ND	+	ND	+	ND	N/A	N/A	+
Spleen	ND	+	ND	+	ND	N/A	N/A	+
