# Supplementary material for: Reverse vaccinology-based identification of a novel surface lipoprotein that is an effective vaccine antigen against bovine infections caused by Pasteurella multocida
Source: PLoS Pathog. 2023 Mar 24;19(3):e1011249. doi: 10.1371/journal.ppat.1011249 (PMC10075479; doi:10.1371/journal.ppat.1011249)
Supplement: S1 Text — (PDF) [file ppat.1011249.s005.pdf]

Supplemental Material:
Protein Sequences of PmSLP from HS causing strains isolated from NVI-Ethiopia.

>NVI_HS12_Sequence_2019
MKYFDTKKSLPVLCSLLITACSGGGGGNNNVPHPPVEKRTVATTQQVAAKPTPPSESLVKRVLNNSENNPPS
RESEKKASQTPPQTDSAKSTIQSTPAVIPDRTNSLTSKKGQIQMSPEWIGKEEKKYAQYSWEHTPESIPVFKLIE
NNQYKYVDDKYFTLESINLNLTKENQVKEGSYQFSLLDSGVYYGHYLSSNDGIHPEYNFVIAFDKNREYTLKDIT
AEYYNSEGFNYAISDRMKGDYIWQVGDVRLFYTNGSVHGEIIEVNDGSKTALFRFENTADRNPNQIVIVPER
DNRHGLSPRGDRMIMDMHFINGSDGEKYKYVVGHGNSDRYYGTLFATKKDKE

>NVI_HS11_Sequence_2019
MKYFDTKKSLPVLCSLLITACSGGGGGNNNVPHPPVEKRTVATTQQVAAKPTPPSESLVKRVLNNSENNPPS
RESEKKASQTPPQTDSAKSTIQSTPAVIPDRTNSLTSKKGQIQMSPEWIGKEEKKYAQYSWEHTPESIPVFKLIE
NNQYKYVDDKYFTLESINLNLTKENQVKEGSYQFSLLDSGVYYGHYLSSNDGIHPEYNFVIAFDKNREYTLKDIT
AEYYNSEGFNYAISDRMKGDYIWQVGDVRLFYTNGSVHGEIIEVNDGSKTALFRFENTADRNPNQIVIVPER
DNRHGLSPRGDRMIMDMHFINGSDGEKYKYVVGHGNSDRYYGTLFATKKDKE

>NVI_HS10_Sequence_2019
MKYFDTKKSLPVLCSLLITACSGGGGGNNNVPHPPVEKRTVATTQQVAAKPTPPSESLVKRVLNNSENNPPS
RESEKKASQTPPQTDSAKSTIQSTPAVIPDRTNSLTSKKGQIQMSPEWIGKEEKKYAQYSWEHTPESIPVFKLIE
NNQYKYVDDKYFTLESINLNLTKENQVKEGSYQFSLLDSGVYYGHYLSSNDGIHPEYNFVIAFDKNREYTLKDIT
AEYYNSEGFNYAISDRMKGDYIWQVGDVRLFYTNGSVHGEIIEVNDGSKTALFRFENTADRNPNQIVIVPER
DNRHGLSPRGDRMIMDMHFINGSDGEKYKYVVGHGNSDRYYGTLFATKKDKE

>NVI_HS7_Sequence_2019
MKYFDTKKSLPVLCSLLITACSGGGGGNNNVPHPPVEKRTVATTQQVAAKPTPPSESLVKRVLNNSENNPPS
RESEKKASQTPPQTDSAKSTIQSTPAVIPDRTNSLTSKKGQIQMSPEWIGKEEKKYAQYSWEHTPESIPVFKLIE
NNQYKYVDDKYFTLESINLNLTKENQVKEGSYQFSLLDSGVYYGHYLSSNDGIHPEYNFVIAFDKNREYTLKDIT
AEYYNSEGFNYAISDRMKGDYIWQVGDVRLFYTNGSVHGEIIEVNDGSKTALFRFENTADRNPNQIVIVPER
DNRHGLSPRGDRMIMDMHFINGSDGEKYKYVVGHGNSDRYYGTLFATKKDKE

>NVI_HS5_Sequence_2019_partial
MSPEWIGKEEKKYAQYSWEHTPESIPVFKLIENNQYKYVDDKYFTLESINLNLTKENQVKEGSYQFSLLDSGVY
YGHYLSSNDGIHPEYNFVIAFDKNREYTLKDITAEYYNSEGFNYAISDRMKGDYIWQVGDVRLFYTNGSVHGE
IIEVNDGSKTALFRFENTADRNPNQIVIVPERDNRHGLSPRGDRMIMDMHFINGSDGEKYKYVVGHGNSDR
YYGTLFATKKDKE

>NVI_HS5_Sequence_2019
MKYFDTKKSLPVLCSLLITACSGGGGGNNNVPHPPVEKRTVATTQQVAAKPTPPSESLVKRVLNNSENNPPS
RESEKKASQTPPQTDSAKSTIQSTPAVIPDRTNSLTSKKGQIQMSPEWIGKEEKKYAQYSWEHTPESIPVFKLIE
NNQYKYVDDKYFTLESINLNLTKENQVKEGSYQFSLLDSGVYYGHYLSSNDGIHPEYNFVIAFDKNREYTLKDIT
AEYYNSEGFNYAISDRMKGDYIWQVGDVRLFYTNGSVHGEIIEVNDGSKTALFRFENTADRNPNQIVIVPER
DNRHGLSPRGDRMIMDMHFINGSDGEKYKYVVGHGNSDRYYGTLFATKKDKE

>NVI_HS3_Sequence_2019
MKYFDTKKSLPVLCSLLITACSGGGGGNNNVPHPPVEKRTVATTQQVAAKPTPPSESLVKRVLNNSENNPPS
RESEKKASQTPPQTDSAKSTIQSTPAVIPDRTNSLTSKKGQIQMSPEWIGKEEKKYAQYSWEHTPESIPVFKLIE
NNQYKYVDDKYFTLESINLNLTKENQVKEGSYQFSLLDSGVYYGHYLSSNDGIHPEYNFVIAFDKNREYTLKDIT
AEYYNSEGFNYAISDRMKGDYIWQVGDVRLFYTNGSVHGEIIEVNDGSKTALFRFENTADRNPNQIVIVPER
DNRHGLSPRGDRMIMDMHFINGSDGEKYKYVVGHGNSDRYYGTLFATKKDKE

>NVI_HS2_Sequence_2019
MKYFDTKKSLPVLCSLLITACSGGGGGNNNVPHPPVEKRTVATTQQVAAKPTPPSESLVKRVLNNSENNPPS
RESEKKASQTPPQTDSAKSTIQSTPAVIPDRTNSLTSKKGQIQMSPEWIGKEEKKYAQYSWEHTPESIPVFKLIE
NNQYKYVDDKYFTLESINLNLTKENQVKEGSYQFSLLDSGVYYGHYLSSNDGIHPEYNFVIAFDKNREYTLKDIT
AEYYNSEGFNYAISDRMKGDYIWQVGDVRLFYTNGSVHGEIIEVNDGSKTALFRFENTADRNPNQIVIVPER
DNRHGLSPRGDRMIMDMHFINGSDGEKYKYVVGHGNSDRYYGTLFATKKDKE

>NVI_HS1_Sequence_2019
MKYFDTKKSLPVLCSLLITACSGGGGGNNNVPHPPVEKRTVATTQQVAAKPTPPSESLVKRVLNNSENNPPS
RESEKKASQTPPQTDSAKSTIQSTPAVIPDRTNSLTSKKGQIQMSPEWIGKEEKKYAQYSWEHTPESIPVFKLIE
NNQYKYVDDKYFTLESINLNLTKENQVKEGSYQFSLLDSGVYYGHYLSSNDGIHPEYNFVIAFDKNREYTLKDIT
AEYYNSEGFNYAISDRMKGDYIWQVGDVRLFYTNGSVHGEIIEVNDGSKTALFRFENTADRNPNQIVIVPER
DNRHGLSPRGDRMIMDMHFINGSDGEKYKYVVGHGNSDRYYGTLFATKKDKE
