# Supplementary material for: Reverse vaccinology-based identification of a novel surface lipoprotein that is an effective vaccine antigen against bovine infections caused by Pasteurella multocida
Source: PLoS Pathog. 2023 Mar 24;19(3):e1011249. doi: 10.1371/journal.ppat.1011249 (PMC10075479; doi:10.1371/journal.ppat.1011249)
Supplement: Attachment — Submitted filename: Response_to_Reviewer_Comments-2023-01-20.pdf [file ppat.1011249.s006.pdf]

Biochemistry
UNIVERSITY OF TORONTO

Thursday, January 19, 2023

Dear PLoS Pathogens Editor,

Thank you and to the reviewers for your consideration of our manuscript (PPATHOGENS-D-22-01655) titled, “Reverse vaccinology-based identification of a novel surface lipoprotein that is an effective vaccine antigen against bovine infections caused by *Pasteurella multocida*”. We appreciate the detailed reviewer comments and have integrated the suggestions towards improving the attached manuscript.

We have paid close attention to revising any statements that add specificity towards the results and the discussion. We additionally appreciate the reviewer who caught the mislabelled statistics in figures 2 and 3, which has been rectified in the updated draft. A statistical analysis section has been added to the Methods section, and the Results section regarding the phylogenetic analysis has been expanded for clarity. The Discussion section has been revised per the comments, including to limit the focus on specific results and expanding the discussion to further include comments on PmSLP-2, the limitations of the current study, and the impact of the study. Additionally, minor changes have been made to the manuscript to ensure readability and consistency throughout. The references have been updated as requested by the reviewers where necessary.

All figure files have been run through PACE to ensure they are suitable for publication and our raw data has been appended as Supplementary Figure 3. Additionally, figures have been removed from the draft manuscript and figure legends have been moved to end of the manuscript, as per PloS Pathogens guidelines.

We appreciate the opportunity to publish this work in PLoS Pathogens and welcome any further feedback on the updated manuscript.

Below is a detailed response to each reviewer comment.

Sincerely,

Trevor Moraes

Trevor F. Moraes (Ph.D.)
Professor, Department of Biochemistry

University of Toronto
MaRS Center, West Tower
661 University Ave., Rm 1613, Toronto, ON M5G 1M1
Email: trevor.moraes@utoronto.ca
Phone: (416) 946-3048; Fax: (416) 946-8228
Web address: http://moraes.biochemistry.utoronto.ca/

Part I - Summary
Please use this section to discuss strengths/weaknesses of study, novelty/significance, general execution and scholarship.

Reviewer #1: Because of the absence of good commercial vaccines against Pasteurella multocida, septicemia and bronchopneumonia caused by this bacterium continues to affect cattle worldwide. This manuscript describes a new P. multocida surface lipoprotein (named PmSLP) and reports the efficacy of this lipoprotein for inducing protection against P multocida infection in mice and cattle. It also reports stability of PmSLP under different storage conditions. The studies reported were well conducted and conclusions are fully supported by the results. The manuscript is well written and easy to follow.

Reviewer #2: I would like to congratulate the authors for conducting such thorough study reflecting multi-institutional and international collaborations. The results suggest that the novel surface lipoprotein could be effectively used to reduce the burden of hemorrhagic septicemia in endemic areas. Hopefully, the authors will demonstrate a reduction of BRD burden with the use of this vaccine in feedlot cattle in the future.

-We thank the reviewers for their time and careful appreciation of this work.

Part II – Major Issues: Key Experiments Required for Acceptance
Please use this section to detail the key new experiments or modifications of existing experiments that should be absolutely required to validate study conclusions.

Generally, there should be no more than 3 such required experiments or major modifications for a "Major Revision" recommendation. If more than 3 experiments are necessary to validate the study conclusions, then you are encouraged to recommend "Reject".

Reviewer #1: No major issues

Reviewer #2: (No Response)

Part III – Minor Issues: Editorial and Data Presentation Modifications
Please use this section for editorial suggestions as well as relatively minor modifications of existing data that would enhance clarity.
Reviewer #1: FYI: The absence of line numbers makes the manuscript difficult to review.
– Apologies for this oversight, line numbers have been added to the resubmitted draft.

Please find below some specific comments to consider.
Introduction:
- 2nd paragraph: in the reviewer's opinion, this paragraph is substandard compared to the rest of the manuscript and should be revised. Please define each abbreviation and avoid starting a sentence with an abbreviation or a number. For example, define HS and BRD.

– This paragraph has been revised, with attention to ensuring all abbreviations are well defined.
- 3rd paragraph: "short duration of protection": add a reference to support your statement. – Added, thank you.
- 5th paragraph: maybe replace " increasing sensitivity" by "increasing scrutiny" – Agreed, this has been edited.
Results section:
2nd paragrah: Bovine are ruminant. Please revise. – We appreciate the reviewer catching this, we meant non-bovine

ruminants. This has been amended.
Page 7, 3rd paragraph: "H229whole bacteria" => "H229 whole bacteria" – thank you!
Figure 2A: How can you get a 20% survival with 4 mice per group? (shouldn't t it be 25%?) Please add the number of mice per group in each figure (i.e, Fig 2, 3, 5, etc. and whenever possible) that will ease the interpretation of the results presented. – Group sizes for the lethal dose determination ranged between 4 to 6 mice per group, with the lowest infectious dose ending with 16.7% survival (1/6 mice), though the authors agree it can be difficult to see on the figures as is. Group sizes have been explicitly defined in each figure legend for clarity for all figure legends where relevant.
Table 2: please move to supplementary material. - Done

Discussion:
Page 21, 1st paragraph: "due to geographical separation of these diseases": cattle in Africa and Asia can suffer from both hemorrhagic septicemia and bronchopneumonia caused by P multocida. Thus, these diseases are not geographically separated. Please revise. – Revised to focus on capsule and PmSLP-type.
Materials and Methods:
Page 22, last paragraph: define LB. – Done.
Page 24, last paragraph: replace "four cattle studies" by "four cattle experiments" to avoid repetition. Please define HI – Done.
Page 25, 2nd paragraph: "the dose used here" => please add a reference. – Dosing studies were done locally by each institution, and not drawn from publications, therefore references are not available.
Reviewer #2: 1.-Please, provide a rationale for not considering PmSLP-2 as part of the vaccines to study. – While the authors of this study certainly consider PmSLP-2 as an attractive vaccine candidate antigen and potentially necessary towards a robust BRD vaccine, inclusion of this antigen was beyond the scope of this current study. A paragraph describing this has been included in the discussion.

2.-Please, provide information about the clinical score used in mice. – Further details have been added to the results section detailing the development of the murine sepsis model.

3.-How were the animals (mice and cattle) housed and handled? Were they the experimental units? Or was there a cage/pen effect? – Further details regarding housing and handling of animals have been added to the methods. Each animal has been considered an independent experimental unit in these studies and no noted cage or pen effect has been seen.

4.-In several plots, the axis read log2 dilution when titre is meant, change to log 2 or log 2 titre as needed. – Thank you, this has been corrected.

5.-There seems to be some mislabeling in figure 2 panels I and J and Figure 3 panel G: check the bars representing the statistical significance. – Thank you for catching, this has been corrected.

6.-Consider support the statements in the section of stability studies (page 24) with references. – Thank you, references have been added to the stability studies in both the methods as well as the results sections.

7.-Consider substituting "highly fatal" in page 3 with "mostly fatal" - This has been reworded as 'frequently fatal'.

8.-I would suggest providing more nuance to the statement that the prophylactic and metaphylactic use of antimicrobials in the cattle industry is directed against P. multocida. In reality, it is against BRD, of which P. multocida is only one of the bacterial agents. – Thank you for this comment, this statement has been reworded to add additional context.

9.-Is there more information about the 18 isolates from ruminants? From what ruminant species? – Yes, this has been further broken down for clarity.

10.-Was the proportion of animals with transient local reactions from the vaccinated groups compared with that of

the control group? -This information has been added.

11.-For how long and at what temperature were the diluted serum samples incubated as part of the ELISA assays? – This had been added to the methods section; samples were incubated at 4C overnight.

12.-In page 5, it is mentioned that PmSLP was present in 112 bovine isolates, but in a previous paragraph it is stated that 65 had PmSLP-1, 11 had PmSLP-2, and 30 had PmSLP-3. What type of PmSLP did the other 6 isolates have? – This has been clarified in text.

13.-The discussion should be substantially modified. The repetition of results should be kept to a minimum and no new results should be presented in this section. No figures or tables should be referenced in the discussion. The discussion of the relevant literature is limited and should be strengthened. There is no discussion of the limitations of the study. A paragraph expanding on the broader impact of this study and how it may benefit further research and development of vaccines against other bacterial agents is warranted. – Thank you for this comment. We have altered the discussion to minimize the repetition of results, and have included further discussion of additional antigen PmSLP-2, as well as additional limitations of this study and the broader impact.

14.-I consider a section of statistical analysis should be added to the methods. – Added, thank you.
